# Etablierung von Pflegekammern in Deutschland – Professionelle Verantwortung und gesellschaftliche Notwendigkeit: Konsentierte gemeinsame Stellungnahme der beiden Arbeitsgruppen „Pflege und Ethik I“ und „Pflege und Ethik II“ in der Akademie für Ethik in der Medizin (AEM) e. V. (Göttingen, Oktober 2021)

**DOI:** 10.1007/s00481-021-00674-7

**Published:** 2021-11-29

**Authors:** Constanze Giese, Andrea Kuhn, Sonja Lehmeyer, Wolfgang Pasch, Annette Riedel, Lutz Schütze, Stephanie Wullf

**Affiliations:** 1 Katholische Stiftungshochschule München, Preysingstraße 95, 81667 München, Deutschland; 2 Hochschule für Wirtschaft und Gesellschaft Ludwigshafen, Ernst-Boehe-Straße 4, 67059 Ludwigshafen, Deutschland; 3 Hochschule Esslingen, Flandernstraße 101, 73732 Esslingen, Deutschland; 4 Fliedner Fachhochschule Düsseldorf, Geschwister-Aufricht-Straße 9, 40489 Düsseldorf, Deutschland; 5 Hochschule Hannover, Ricklinger Stadtweg 120, 30625 Hannover, Deutschland; 6 Universität Hildesheim, Universitätsplatz 1, 31141 Hildesheim, Deutschland

## Relevanz und Hintergrund der Stellungnahme

Pflege umfasst die eigenverantwortliche Versorgung und Betreuung – allein oder in Kooperation mit anderen Berufsangehörigen – von Menschen aller Altersgruppen, von Familien oder Lebensgemeinschaften, sowie von Gruppen und sozialen Gemeinschaften, ob krank oder gesund, in allen Lebenssituationen (Settings). Pflege schließt die Förderung der Gesundheit, die Verhütung von Krankheiten und die Versorgung und Betreuung kranker, behinderter und sterbender Menschen ein. Weitere Schlüsselaufgaben der Pflege sind Wahrnehmung der Interessen und Bedürfnisse (Advocacy), Förderung einer sicheren Umgebung, Forschung, Mitwirkung in der Gestaltung der Gesundheitspolitik sowie im Management des Gesundheitswesens und in der Bildung.^1^

Der Heilberuf Pflege mit seinem am Zentralwert Gesundheit ausgerichteten gesellschaftlichen Mandat ist mit definierten Vorbehaltsaufgaben betraut und gesetzlich legitimiert. Pflegefachpersonen als Vertreter*innen der Berufsgruppe obliegt eine spezifische professionelle Verantwortung. Um ihrem gesetzlich definierten Auftrag in beruflicher Autonomie und Eigenverantwortung verlässlich zu entsprechen, verpflichten sich Pflegefachpersonen einem gemeinsam konsentierten und verbindlichen Berufsethos, das über Landesgrenzen hinaus weltweit akzeptiert ist: Der ICN-Ethikkodex ist international für 20 Mio. Pflegefachpersonen gültig und von den nationalen Kammern oder berufsständischen Vertretungen in 130 Ländern ratifiziert^2^.

Gesellschaftliche Entwicklungen und daran gebundene Innovations- und Transformationsbedarfe in der Pflege machen auch in Deutschland die dauerhafte Etablierung von Pflegekammern unausweichlich. Das Fehlen einer berufsständischen Vertretung der Pflege im Bereich der sozialrechtlichen Selbstverwaltung des Gesundheitswesens stellt ein Risiko für die Versorgungssicherheit und Versorgungsqualität der Bevölkerung dar. Pflegekammern derzeit immer noch in Frage zu stellen, erscheint angesichts der Herausforderungen im Pflege- und Gesundheitswesen geradezu paradox. Die Aufgaben von Pflegekammern werden nachfolgend hinsichtlich ihrer gesellschaftlichen Relevanz und ihres professionsethischen Auftrags in Anlehnung an den Ethikkodex des International Council of Nurses (ICN) erläutert.

## Pflegende und ihre Mitmenschen

Die zentrale Funktion von Pflegekammern besteht darin, die pflegerische Versorgung der Bevölkerung gemäß des jeweils aktuellen Stands des Wissens abzusichern. Pflegekammern entwickeln Handlungsempfehlungen und Richtlinien, mit denen aktuelles Pflegewissen und professionsethische Standards auf dem je neuesten Stand allen Pflegefachpersonen beständig zugänglich gemacht werden. Dies umfasst auch Verfahren der Entscheidungsfindung für (inter-)professionelle Versorgungsteams aller Settings und Organisationen und für das professionelle Arbeitsbündnis zwischen Pflegefachpersonen und Menschen mit Pflegebedarf. Pflegekammern erhöhen dadurch strukturell wie handlungspraktisch den Schutz vulnerabler Personen und Gruppen innerhalb des Gesundheitswesens. Sie tragen maßgeblich zu einer menschenrechtssensiblen wie ethisch fundierten pflegerischen Versorgung der Bevölkerung bei. Pflegekammern unterstützen sichere und gesundheitsförderliche Bedingungen in allen Bereichen des Gesundheitswesens. Ausdrücklich treten sie für die gerechte Verteilung von Ressourcen, den universellen Zugang zur Gesundheitsversorgung, eine angemessene Information der Menschen mit Pflegebedarf sowie den vertraulichen Umgang mit persönlichen Daten ein.

Pflegekammern tragen maßgeblich dazu bei, eine vertrauenswürdige und qualitativ hochwertige pflegerische Versorgung der Bevölkerung zu gewährleisten.

## Pflegende und die Berufsausübung

Jede Pflegefachperson trägt als Angehörige des Heilberufes Pflege persönlich Verantwortung für die Ausübung professioneller Pflege, darüber sind die Pflegefachpersonen jederzeit rechenschaftspflichtig. Pflegekammern lassen Pflegefachpersonen mit diesem Anspruch nicht allein: Die Berufsordnung legt Aufgaben und Verantwortungsbereiche fest, Mitglieder berufen sich auf ihr normiertes Berufsrecht und fordern es ein.

Kontinuierliches Lernen hält berufliche Kompetenzen auf dem aktuellen Stand professionellen Wissens. Pflegekammern fördern dies über Bildungsangebote, Medien, Konferenzen u. a. und führen den Nachweis über Fort- und Weiterbildungsregister. Pflegekammern unterstützen durch Qualitätsstandards und Qualifikationsangebote die Anwendung neuen Wissens, neuer Methoden und neuer Technik. Vereinbarte Qualitätsindikatoren sichern die Würde, die Rechte und die Sicherheit von Menschen mit Pflegebedarf und die Pflegequalität ab. Fachlich und persönlich korrektes Verhalten stärken das Vertrauen in den Berufsstand.

Pflegefachpersonen und Pflegekammern stehen gemeinsam in der Verantwortung, die Fähigkeit zur Berufsausübung zu erhalten und Beeinträchtigungen entgegenzuwirken. Durch ihr systematisches und kontinuierliches Eintreten für adäquate und gesundheitsförderliche Arbeitsbedingungen und das Angebot unterstützender Dienstleistungen für Pflegefachpersonen dient die Kammer ihren Mitgliedern und der Gesellschaft gleichermaßen. Darin erweisen sich die Verwobenheit berufs-, organisations- und sozialethischer Implikationen einer Verkammerung und deren hohe gesellschaftliche Relevanz.

Pflegekammern setzen sich für Rahmenbedingungen ein, die es Pflegefachpersonen ermöglichen, unter Wahrung ihrer professionellen Integrität ihrem beruflichen Auftrag nachzukommen.

## Pflegende und die Profession

Pflegekammern erfüllen zentrale Aufgaben in der Befähigung jeder einzelnen Pflegefachperson, ihrer Verantwortung für die Ausübung der Pflege vor Ort gerecht zu werden. Durch ihre Zuständigkeit für die berufliche Fort- und (Fach‑)Weiterbildung sorgen die Kammern unabhängig von ökonomischen Interessen dafür, dass alle aktuellen Wissensbestände und Kompetenzen adressiert werden, die Pflegefachpersonen benötigen, um eine professionelle Pflegebeziehung auf der Basis aktueller und kritisch reflektierter fachlicher Evidenz sowie unter Einbezug der Patient*innenrechte im Sinne einer grund- und menschenrechtlichen Fundierung zu gestalten. Zudem binden Pflegekammern ihre Mitglieder in aktuelle, sie betreffende Diskurse ein. Sie informieren über zentrale rechtliche Neuerungen und sorgen dafür, dass pflegerelevante Erkenntnisse zeitnah in die Aus‑, Fort- und (Fach‑)Weiterbildung einfließen.

Pflegekammern gewährleisten die Aufnahme von Bildungsinhalten, die Pflegefachpersonen über eine direkte Verwertbarkeit in der Berufspraxis hinaus befähigen, ihre Verantwortung für den Pflegeprozess wahrzunehmen und die dafür erforderlichen Arbeitsbedingungen nachhaltig mitzugestalten. So sind Pflegekammern zugleich Ansprechpartner für ihre Mitglieder angesichts ethisch herausfordernder Arbeitsbedingungen und strukturell bedingter Versorgungsprobleme.

Lediglich Pflegekammern können frei von anderen Interessen und legitimiert durch die Mitgliedschaft aller Pflegefachpersonen ihre Verantwortung für die Entwicklung der Profession gemäß ihres gesellschaftlichen Auftrags in international anschlussfähiger Weise übernehmen.

## Pflegende und ihre Kolleginnen

Pflegekammern repräsentieren die transparent verfasste und formal legitimierte Vertretung der Pflege in der Selbstverwaltung des Gesundheitswesens. Sie bringen die pflegespezifische Expertise im Sinne des pflegeprofessionellen gesellschaftlichen Mandats in die jeweiligen Gremien ein und lancieren die interprofessionelle Zusammenarbeit mit den anderen Standesvertretungen und Leistungspartnern. Sie sichern den Informationsfluss zwischen Gremien und Kammermitgliedern ab, gestalten intraprofessionelle Diskurse und erarbeiten berufsgruppenspezifische Positionierungen. Pflegekammern fördern das ethische Verhalten ihrer Mitglieder und stärken die ethische Kompetenz einschließlich der ethischen Sprach‑, Diskurs- und Handlungsfähigkeit durch ethische Beratungs- und Bildungsangebote. Dies dient der Gestaltung der (inter‑)professionellen, ethisch fundierten Zusammenarbeit in der Berufspraxis wie auf gesundheitspolitischer Ebene, dies fördert Gleichbehandlung, soziale Gerechtigkeit wie auch Chancengerechtigkeit.

Pflegekammern tragen dazu bei, dass Pflegefachpersonen ihrem gesellschaftlichen und professionellen Auftrag – auch hinsichtlich des ethischen Begründens, Verhaltens und Handelns – im Zusammenspiel (inter-)professioneller Versorgungsteams aber auch im Kontext nachhaltiger Entwicklungsziele aktiv nachkommen.

## Zusammenfassend

Die Forderung nach einer berufsständischen Vertretung in Form von Pflegekammern ist – wie deutlich wurde – explizit kein Selbstzweck einer sich fortschreitend professionalisierenden Berufsgruppe. Die Erfahrungen der COVID-19-Pandemie haben dies eindrücklich aufgezeigt^3^.

Gut funktionierende Pflegekammern sind für die gesundheitliche und pflegerische Versorgungssicherheit der Bevölkerung von zentraler Bedeutung. Die Aufgaben- und Verantwortungsbereiche von Pflegekammern auf Landes- und auf Bundesebene können ausschließlich von einer berufsständisch selbstverwalteten Pflege kompetent übernommen und im Sinne des bestehenden gesellschaftlichen Mandats ausgefüllt werden. Für Pflegefachpersonen als Vertreter*innen des Heilberufs Pflege stellt eine verpflichtende Kammermitgliedschaft somit keine – wie aktuell suggeriert und in Teilen praktiziert – an- oder abzulehnende Wahloption dar^4^. Sie ist vielmehr eine Verpflichtung, der es nachzukommen und eine Verantwortung, welche es auszufüllen gilt.

Diese Stellungnahme soll zu einer informierten Auseinandersetzung mit dem Thema Pflegekammern seitens der Mitglieder der Profession Pflege selbst sowie aller anderen Disziplinien und Heilberufe innerhalb der Akademie für Ethik in der Medizin – und darüber hinaus – beitragen. Gleichsam versteht sie sich als Impuls zur Versachlichung der fortdauernden, von vielfältigen Interessen geleiteten Debatte um Pflegekammern aus einer ethisch fokussierten Perspektive^5^.

## Footnotes

1 DBfK, ÖGKV & SBK (o.J.) Konsentierte deutsche Übersetzung der Definition der Pflege. https://www.dbfk.de/media/docs/download/Allgemein/Definition-der-Pflege-ICN-deutsch.pdf [zugegriffen: 05. Okt. 2021].

2 ICN-Ethikkodex für Pflegende (2012) (deutschsprachige Übersetzung). https://www.dbfk.de/media/docs/download/Internationales/ICN_Code-of-Ethics_DE_WEB_clean.pdfhttps://www.dbfk.de/de/shop/artikel/ICN-Ethikkodex-fuer-Pflegende.php. [Zugegriffen: 05. Okt. 2021].

3 vgl. hierzu das Diskussionspapier der beiden Arbeitsgruppen vom 12.05.2020. https://www.aem-online.de/fileadmin/user_upload/2020_05_12_Pflegeethische_Reflexion_Papier.pdf [zugegriffen: 05. Okt. 2021].

4 Die Diskussion um die Errichtung und den Erhalt von Landespflegekammern verläuft in den Bundesländern (berufs-)politisch äußerst kontrovers. Zum aktuellen Sachstand der Entwicklungen auf Länderebene vgl. hierzu https://www.aok-verlag.info/de/news/Errichtung-von-Pflegekammern-in-den-einzelnen-Bundeslaendern/28/ [05. Okt. 2021].

5 vgl. hierzu die frühere Stellungnahme der AG „Pflege und Ethik I“ (Mai 2013). Abrufbar unter: https://www.ksh-muenchen.de/fileadmin/user_upload/professors/16571/Publications/2292.pdf [zugegriffen: 05. Okt. 2021].

